# Peritoneal free air due to evacuation of pneumobilia in blunt abdominal trauma

**DOI:** 10.4103/0974-2700.70777

**Published:** 2010

**Authors:** Isaac Howley, Shea C Gregg, Daithi S Heffernan, Charles A Adams

**Affiliations:** Department of Surgery, Rhode Island Hospital and the Warren Alpert School of Medicine, Brown University, Providence, RI 02903, USA

**Keywords:** Pneumobilia, pneumoperitoneum, trauma

## Abstract

Pneumobilia is mostly observed on computed tomography (CT) following surgical biliary-enteric anastomosis and biliary manipulation through endoscopic procedures. Although pneumobilia can be seen in pathological conditions, post-surgical pneumobilia is typically not associated with morbidity. In the present article, we report a case in which blunt abdominal trauma led to the evacuation of pre-existing pneumobilia causing pneumoperitoneum. Given that the subsequent laparotomy proved to be non-therapeutic, this report adds to the few cases of intra-peritoneal free air not helped by surgical intervention.

## INTRODUCTION

Pneumoperitoneum in blunt abdominal trauma is typically associated with the rupture of a hollow viscus and remains a surgical emergency. We present a case of pneumoperitoneum that was not helped by therapeutic laparotomy due to its unique pathophysiology arising from hepatic fracture and pneumobilia.

## CASE REPORT

A 65-year-old woman presented as an unrestrained driver in a frontal impact motor vehicle crash. Upon arrival to our emergency department, her pulse was 100, blood pressure was 125/76 mmHg, Glasgow Coma Score was 15, and she complained of right upper quadrant pain. Physical examination revealed previous surgical scars, moderate abdominal distention, and right upper quadrant localized tenderness. The secondary survey was negative for additional examination findings.

**Figure 1:**
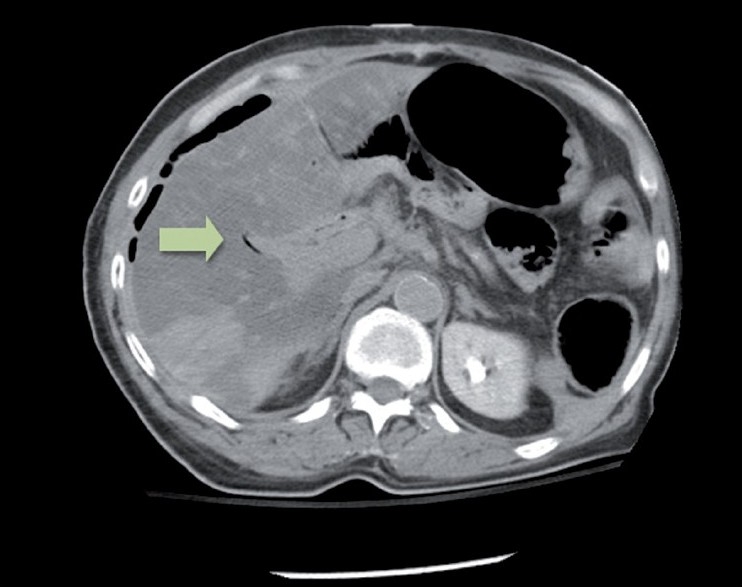
CT scan of the abdomen prior to the patient’s current admission demonstrating pneumobilia but no intraperitoneal free air

The patient’s past medical history was significant for chronic pancreatitis, which was refractory to medical management. Due to her severe pain, the patient underwent a cholecystectomy with a combined Roux-en-Y longitudinal pancreaticojejunostomy and hepaticojejunostomy, 14 months prior to the current presentation. As a result of this biliary-enteric anastomosis, the patient was noted to have asymptomatic pneumobilia [Figure 1]. Additional medical/surgical history included a 30-foot fall from her rooftop, 3 months prior to the current presentation, which necessitated a splenectomy due to hemorrhagic shock.

**Figure 2:**
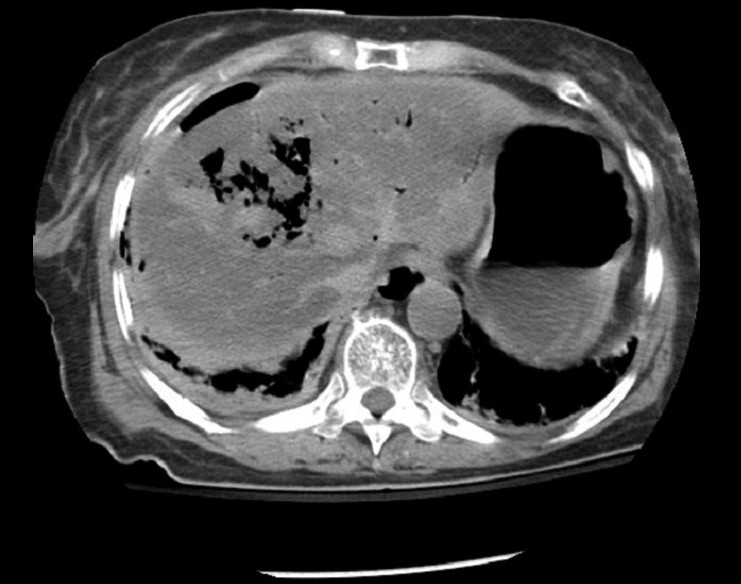
CT scan of the abdomen on patient presentation demonstrating pneumobilia and pneumoperitoneum anterior to the liver

Laboratory examination revealed the following: WBC 15.3 × 10^3^/mm^3^, hemoglobin 11.3 g/dL, Aspartate aminotransferase (AST) 1698 U/L, alanine transaminase (ALT) 783 U/L, alkaline phosphatase 376 U/L, total bilirubin 1.2 mg/dL, direct bilirubin 0.4 mg/dL, albumin 3.1 g/dL, and troponin 0.19 ng/mL. Her chest X-ray, head computerized tomography (CT), and cervical spine CT examinations were negative for any acute injuries. A CT of her abdomen and pelvis at this admission showed a large amount of pneumobilia, moderate amount of peritoneal free air around the liver, perihepatic free fluid, and an American Association for the Surgery of Trauma (AAST) grade II liver laceration[1] to the dome of the right lobe of the liver [Figure 2]. There was no evidence for pneumothorax on the CT scan. Given the patient’s mechanism, abdominal tenderness, and CT findings, our major concerns included hollow viscus injury. As such, we proceeded to the operating room for an exploratory laparotomy.

A midline incision incorporated the scar from the patient’s previous abdominal surgery. Extensive abdominal adhesions were encountered and care was taken not to injure the abdominal viscera while efficiently attempting to access the peritoneal cavity. Inferior to the umbilicus, a moderate amount of hemoperitoneum was encountered; however, no enteric contents were appreciated. There were no injuries on any intestinal surface to explain the free air. The Roux limb from the patient’s hepatico- and pancreaticojejunostomies was dissected free; however, it too had no evidence of injury or perforation. Upon examination of the right upper quadrant, pockets of free air surrounded the liver. A minimally bleeding liver laceration was encountered on the dome of the liver, just anterior to the inferior vena cava. Upon further examination, air was tracking under the liver capsule into the right lobe. When the liver was manipulated, small air bubbles arose from within the parenchyma and exited through the laceration. Hemostasis was achieved with FloSeal (Baxter, Deerfield, IL, USA) and Surgicel (Ethicon, Cincinnati, OH, USA). The abdomen was copiously irrigated and closed after leaving a 10 mm flat Jackson-Pratt drain in the upper right quadrant adjacent to the lateral aspect of the liver.

**Figure 3:**
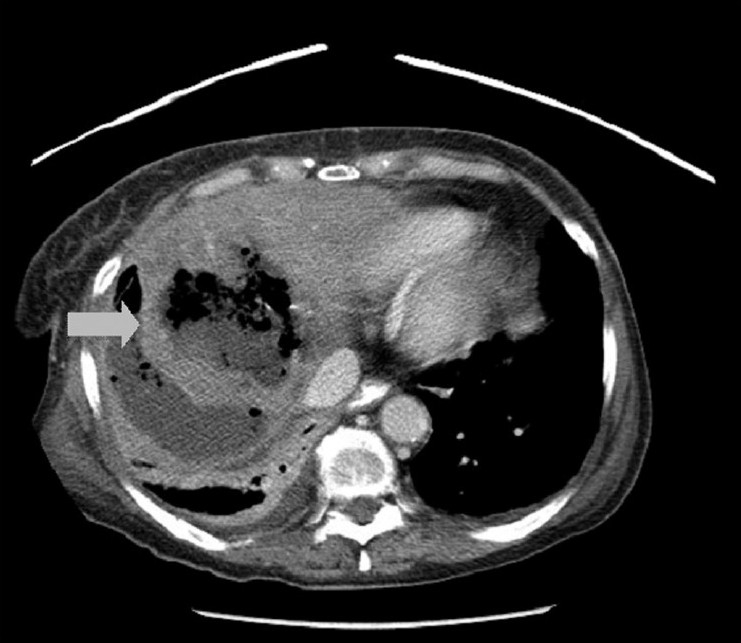
CT scan of the abdomen on post-operative day 9 demonstrating intra-hepatic abscess

Post-operatively, the patient recovered in the Trauma Intensive Care Unit for 3 days before transfer to a general surgical floor. Her post-injury course was complicated by the development of fevers, leukocytosis, and hypotension on post-operative day 9 which necessitated transfer back to the Intensive Care Unit. A CT of the abdomen/pelvis showed a 6.9 × 8.1 cm hepatic abscess [Figure 3]. This was treated with antibiotics and percutaneous drainage yielding fluid that grew mixed enteric flora by culture. The remainder of her hospital course was uncomplicated. She was transferred back to a general surgical floor on post-injury day 18, and discharged from the hospital the following day.

**Figure 4:**
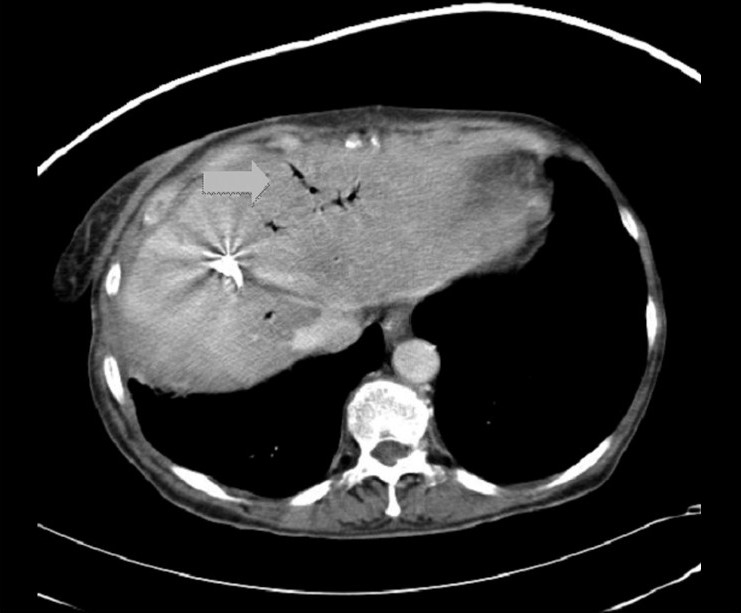
CT of the abdomen prior to drain removal demonstrating both healing of the liver laceration and resolution of the intra-hepatic infection; however, pneumobilia persists

The patient was seen in follow-up on several occasions and has continued to do well. A follow-up CT of the abdomen prior to drain removal demonstrated both healing of the liver laceration and resolution of the intra-hepatic infection [Figure 4].

## DISCUSSION

Pneumobilia is an uncommon radiographic finding and may occur secondary to surgical biliary-enteric anastomosis or biliary manipulation through endoscopic procedures. In the trauma literature, it has been described as a rare consequence of blunt abdominal injury.[2–4] In the present case, our patient sustained enough force to her abdomen to not only exacerbate her pre-existing pneumobilia, but also induce liver fracture and pneumoperitoneum without hollow viscus perforation. Given this unique finding, the pathophysiology comes into question. The normal pathophysiology of intraperitoneal free air – rupture of hollow viscus – was not encountered in our patient. Thus, possible mechanisms may include 1) free air leaked out of the lacerated liver following the initial injury and/or 2) the initial impact created enough intraluminal pressure to cause forceful retrograde evacuation of bowel contents through the Roux limb into the biliary system, thus causing capsular rupture. In support of delayed leakage, Cheynel *et al*. simulated hepatic frontal deceleration injuries using cadaveric livers and found that rotation around the axis of the inferior vena cava and differential motion of the right and left lobes caused lacerations in the caval area.[5] In congruence with this report, our patient’s injury was in a location that would be considered “expected”, given the patient’s initial mechanism of injury. What remains to be explained is why pneumoperitoneum has not been reported in the past with such lacerations. With regard to the second theory, Gering *et al*. proposed that traumatic pneumobilia occurred due to increased duodenal intraluminal pressure forcing bowel gas through the sphincter of Oddi.[3] Our patient’s previous hepatojejunostomy had removed the sphincter of Oddi, thus removing any resistance to further bowel gas entering the biliary tree. Without this resistance, a sudden increase in intraluminal pressure can be hypothesized to exert enough force in the biliary tree to ultimately lead to rupture of the capsule. This is especially compelling given the fact that the patient subsequently developed an intra-hepatic abscess that grew out GI based flora. In summary, it is likely that both these mechanisms contributed to the patient’s unique presentation.

While our patient did not receive any therapeutic benefit from our exploratory laparotomy, it was required given the free intraperitoneal air following traumatic mechanism and abdominal tenderness. From before the time of Pringle until the early 1990s, surgical intervention was considered almost always necessary in blunt hepatic trauma. Richardson *et al*. reported that non-surgical treatment of blunt liver trauma increased from 0% of cases in 1985–1989 to 65% in 1995–1999. The same period saw a consistent decline in both total and liver-related death rates.[6] In the absence of free air, our patient would have not required operative intervention and might have been successfully managed nonoperatively. In theory, triple-contrast CT may have been used to demonstrate lack of bowel perforation; however, given that free air was present and that operative management would have been chosen regardless of imaging result, the added contrast, time, and cost necessary to perform such an examination was not going to change or augment the patient’s care.

Although abdominal free air in blunt abdominal trauma is generally associated with hollow viscus perforation, other causes have been described. Polychronidis *et al*. from Greece described a case of right-sided blunt thoracoabdominal trauma in which a hemidiaphragm rupture with hepatic herniation led to copious pneumoperitoneum as demonstrated by CT.[7] Ferrera and Chan have similarly described pneumoperitoneum resulting from diaphragm rupture.[8] Interestingly, Polychronidis’ patient was noted to have a normal abdominal examination, whereas Ferrera and Chan’s examination was limited by sedation. In contrast to these reports which required operative intervention, other traumatic cases of free air have been described in the literature, which did not benefit from laparotomy. Shiomi *et al*. reported a case in which a motorcycle accident resulted in urogenital lacerations and a handlebar hernia; atmospheric air dissected via the subcutaneous tissue from the lacerations to the hernia and into the peritoneal cavity.[9] Hamilton *et al*. from the University of Toronto reported a series of 118 consecutive abdominal CT scans for blunt trauma in which seven patients had free abdominal air.[10] Of these seven patients, none had bowel injuries; but all had received chest tubes, to which the authors attributed the CT findings. Although such rare cases may occur, we would not advocate foregoing laparotomy in the presence of free air in acute trauma.

As shown in Figures 2 and 3, hepatic abscess formation in our patient seemed to result from bacterial seeding during the initial impact. In general, abscess formation is a rare complication of non-operative management for blunt hepatic injury, occurring in 0–0.6% of cases.[11,12] Operative treatment of blunt hepatic injury, however, has been associated with an intra-abdominal abscess rate of approximately 9%.[13,14] In a series of 389 patients treated non-operatively for isolated hepatic trauma, 6 developed abscesses, as compared with 15 patients out of the 173 patients who received operative treatment (*P* < 0.001).[15] These non-randomized data do not prove that operative treatment is a causative factor in abscess formation; this could be explained by the development of devascularized tissue and its secondary infection both from the injury itself and the usually non-anatomic surgical debridement that occurs during exploration. Regarding their management, several large retrospective studies have established the safety and efficacy of percutaneous drainage for unilocular abscesses.[16–19] Fortunately, our patient responded successfully to this approach despite the unconventional mechanism.

To our knowledge, this is the first case in the English literature of what appears to have been pneumoperitoneum originating from preexisting pneumobilia following a traumatic mechanism. Pneumobilia is an uncommon finding, although its prevalence may increase with biliary-enteric drainage procedures.

## CONCLUSION

The patient reported here presented with a rare cause of traumatic pneumoperitoneum that did not receive therapeutic benefit from surgical exploration. In retrospect, this scenario could have been managed non-operatively, however, this was not an appropriate option given the patient’s traumatic mechanism and degree of abdominal tenderness on presentation. In our current age of reliance on CT scan, we must continue to be mindful of a differential diagnosis in our utilization of highly sensitive imaging technology.

## References

[1] Moore EE, Shackford SR, Pachter HL, McAninch JW, Browner BD, Champion HR (1989). Organ injury scaling: spleen, liver, and kidney. J Trauma.

[2] Barnes SL, Badrudduja M, Bernard AC, Boulanger BR (2006). Pneumobilia after blunt trauma: a self-limited condition?. J Trauma.

[3] Gering SA, Foster MA, Harnisch MC, McNeil JJ (2001). Traumatic pneumobilia: case report. J Trauma.

[4] Thompson RJ, Irwin T (2007). Pneumobilia following blunt abdominal trauma. Ir J Med Sci.

[5] Cheynel N, Serre T, Arnoux PJ, Baque P, Benoit L, Berdah SV (2006). Biomechanic study of the human liver during a frontal deceleration. J Trauma.

[6] David Richardson J, Franklin GA, Lukan JK, Carrillo EH, Spain DA, Miller FB (2000). Evolution in the management of hepatic trauma: a 25-year perspective. Ann Surg.

[7] Polychronidis A, Bounovas A, Didilis B, Perente S, Simopoulos C (2001). Intraperitoneal air in the diagnosis of blunt diaphragmatic rupture. J Cardiovasc Surg (Torino).

[8] Ferrera PC, Chan L (1999). Tension pneumoperitoneum caused by blunt trauma. Am J Emerg Med.

[9] Shiomi H, Hase T, Matsuno S, Izumi M, Tatsuta T, Ito F (1999). Handlebar hernia with intra-abdominal extraluminal air presenting as a novel form of traumatic abdominal wall hernia: report of a case. Surg Today.

[10] Hamilton P, Rizoli S, McLellan B, Murphy J (1995). Significance of intra-abdominal extraluminal air detected by CT scan in blunt abdominal trauma. J Trauma.

[11] Malhotra AK, Fabian TC, Croce MA, Gavin TJ, Kudsk KA, Minard G (2000). Blunt hepatic injury: a paradigm shift from operative to nonoperative management in the 1990s. Ann Surg.

[12] Pachter HL, Knudson MM, Esrig B, Ross S, Hoyt D, Cogbill T (1996). Status of nonoperative management of blunt hepatic injuries in 1995: a multicenter experience with 404 patients. J Trauma.

[13] Goins WA, Rodriguez A, Joshi M, Jacobs D (1990). Intra-abdominal abscess after blunt abdominal trauma. Ann Surg.

[14] Fabian TC, Croce MA, Stanford GG, Payne LW, Mangiante EC, Voeller GR (1991). Factors affecting morbidity following hepatic trauma. A prospective analysis of 482 injuries. Ann Surg.

[15] Hsieh CH (2002). Comparison of hepatic abscess after operative and nonoperative management of isolated blunt liver trauma. Int Surg.

[16] Cheng HC, Chang WL, Chen WY, Kao AW, Chuang CH, Sheu BS (2008). Long-term outcome of pyogenic liver abscess: factors related with abscess recurrence. J Clin Gastroenterol.

[17] Chung YF, Tan YM, Lui HF, Tay KH, Lo RH, Kurup A (2007). Management of pyogenic liver abscesses - percutaneous or open drainage?. Singapore Med J.

[18] Ferraioli G, Garlaschelli A, Zanaboni D, Gulizia R, Brunetti E, Tinozzi FP (2008). Percutaneous and surgical treatment of pyogenic liver abscesses: observation over a 21-year period in 148 patients. Dig Liver Dis.

[19] Hope WW, Vrochides DV, Newcomb WL, Mayo-Smith WW, Iannitti DA (2008). Optimal treatment of hepatic abscess. Am Surg.

